# Incidence trends of surgical complications after oesophagectomy for oesophageal cancer: a population-based, nationwide cohort study in Finland over 30 years

**DOI:** 10.1186/s12957-025-03709-1

**Published:** 2025-02-18

**Authors:** Ville E.J. Sirviö, Jari V. Räsänen, Olli Helminen, Mika Helmiö, Heikki Huhta, Raija Kallio, Vesa Koivukangas, Arto Kokkola, Simo Laine, Elina Lietzen, Sanna Meriläinen, Vesa-Matti Pohjanen, Tuomo Rantanen, Ari Ristimäki, Juha Saarnio, Eero Sihvo, Vesa Toikkanen, Tuula Tyrväinen, Mikko Uimonen, Antti Valtola, Joonas H. Kauppila

**Affiliations:** 1 Department of Oesophageal and General Thoracic Surgery, Helsinki University Hospital, University of Helsinki, Haartmaninkatu 4 PL 340, Helsinki, 00029 HUS Finland; 2 Surgery Research Unit, Oulu University Hospital, University of Oulu, Oulu, Finland; 3 The Division of Digestive Surgery and Urology, Turku University Hospital and University of Turku, Turku, Finland; 4 Department of Oncology and Radiotherapy, Oulu University Hospital, Oulu, Finland; 5 Department of Surgery, University of Helsinki, Helsinki University Hospital, Helsinki, Finland; 6 Cancer and Translational Medicine Research Unit, Medical Research Center Oulu, University of Oulu and Oulu University Hospital, Oulu, Finland; 7 Department of Surgery, University of Eastern Finland, Kuopio University Hospital, Kuopio, Finland; 8 Department of Pathology and HUS Diagnostic Center, University of Helsinki and Helsinki University Hospital, Helsinki, Finland; 9 Applied Tumor Genomics Research Program, Research Programs Unit, University of Helsinki, Helsinki, Finland; 10 Department of Surgery, Central Finland Central Hospital, Jyväskylä, Finland; 11 Department of Cardiothoracic Surgery, Heart Center, Tampere University Hospital, University of Tampere, Tampere, Tampere, Finland; 12 Department of Gastroenterology and Alimentary Tract Surgery, Tampere University Hospital, Tampere, Finland; 13 Upper Gastrointestinal Surgery, Department of Molecular Medicine and Surgery, Karolinska Institutet, Stockholm, Sweden

**Keywords:** Oesophageal cancer, Oesophagectomy, Surgical complications, Morbidity, Mortality, Nationwide cohort

## Abstract

**Background:**

Knowledge on the incidence of surgical complications after oesophagectomy for oesophageal cancer in nationwide practice is scarce. The aim of this study was to assess complication trends after oesophagectomy in a nationwide, population-based, unselected cohort.

**Methods:**

All patients undergoing oesophagectomy for oesophageal cancer in Finland in 1987–2016 were included. All complications defined by the Esophagectomy Complications Consensus Group (ECCG) were reported in three 10-year periods. Chi-square test and Kruskal-Wallis test were used to compare outcomes in these periods.

**Results:**

A total of 1493 patients were included. From 1987 to 1996 to 2007–2016, improvements were seen in the rate of major complications (49% vs. 43%, *p* = 0.039), length of hospital stay (19 vs. 14 days, median, *p* < 0.001), length of ICU-stay (3 vs. 2 days, median, *p* < 0.001) and 90-day mortality (17.9% vs. 5.4%, *p* < 0.001), while pneumonia (16% vs. 23%, *p* = 0.029) and anastomotic leak (8% vs. 12% in total leaks, *p* = 0.006 for type II leak) increased. The most common complications were pneumonia, pleural effusion requiring drainage (16% vs. 23%, *p* = 0.080), atrial dysrhythmia (16% vs. 15%, *p* = 0.464) and anastomotic leak. The most common complication categories defined by ECCG were pulmonary (36% vs. 42%, *p* = 0.151) and gastrointestinal (21% vs. 23%, *p* = 0.398) complications.

**Conclusions:**

This study reports high postoperative morbidity after oesophagectomy in nationwide practice. Mortality has significantly improved during the years, and it appears that patients who would have died earlier, can now be rescued. While the relative occurrence of complications has remained constant, overall morbidity has decreased as the more severe outcomes have decreased.

## Introduction

Oesophageal cancer is the 6th most common cause of cancer mortality worldwide [1]. In locally advanced disease, neoadjuvant treatment combined with oesophagectomy is the gold-standard approach in curatively intended treatment [2]. Oesophagectomy is a highly demanding intervention both for the patient and the surgeon, characterized by high postoperative morbidity.

In the absence of a standardized framework for reporting complications after oesophagectomy, the Esophagectomy Complications Consensus Group (ECCG) platform was created in 2015 to facilitate comparison and assessment of quality improvement efforts [3]. Since then, only one country has reported nationwide data on complications after oesophagectomy adhering to the ECCG classification: A Dutch study reported a 65% nationwide complication rate in the Dutch Upper gastrointestinal Cancer Audit (DUCA) during 2016–2017, as opposed to the 59% found in the initial ECCG study [4, 5]. An international study reported a 64% complication rate in a 9 month sample of oesophagectomies performed across 137 hospitals in 41 countries [6]. No other large-scale reports on complications using the ECCG classification exist.

Postoperative complications are associated with poorer short- and long-term outcomes [7–10], yet knowledge on the incidence of complications after oesophagectomy in a nationwide setting is scarce. Thus, the safety of treatment provided to patients in day-to-day practice is not well known, and further national reporting is needed. Care has evolved with better patient selection, improved imaging and staging, routine neoadjuvant treatment, minimally invasive technique and arguably centralization. However, subsequent developments in complication trends are not well known, as no long-term studies assessing complication rates after oesophagectomy exist.

The aim of this study was to assess the incidence trends of complications in patients undergoing oesophagectomy for oesophageal cancer in Finland during a 30-year period from 1987 to 2016.

## Methods

### Study design

This study was a nationwide Finnish, population-based, retrospective cohort study.

### Data sources

For this study, patient data were retrieved from the Finnish National Esophago-Gastric Cancer Cohort (FINEGO). The cohort includes all patients undergoing oesophagectomy for oesophageal cancer in Finland in 1987–2016, identified through well validated and complete national registries [11, 12]. National registries provided basic patient information, such as age, sex and comorbidity, and clinical variables were retrieved from patient records from individual hospitals and reviewed by expert thoracic- and upper gastrointestinal surgeons. All unique surgical complications were classified according to the ECCG framework and graded for severity of the most serious complication according to the Clavien-Dindo (CD) classification. Comorbidities were defined according to the modified Charlson Comorbidity Index (CCI) [13]. Clinical variables were collected using pre-defined online forms [11]. Mortality data was provided by the 100% complete registry of Statistics Finland [14]. 

### Outcomes

The primary outcome was the occurrence of any surgical complication during 90 days after oesophagectomy. The secondary outcomes were complication categories defined by ECCG, major complications, reoperations, length of ICU stay, length of hospital stay and 90-day mortality.

Complications and complication categories were defined according to the ECCG framework. Major complications were defined as CD IIIa or higher. Reoperations were defined as surgical interventions done in the OR with or without general anesthesia, excluding endoscopic interventions.

### Statistical analysis

Patient and tumor characteristics were presented as frequencies and percentages. Postoperative outcomes were assessed in 3 calendar periods: 1987–1996, 1997–2006 and 2007–2016.

The division into these three periods was determined by changes in practice and cancer incidence. Minimally invasive oesophagectomy and neoadjuvant treatment were implemented in Finland in 2007 and 2005–2007, marking the start of the last period. During the 1990s, the incidence of oesophageal adenocarcinoma started to rise swiftly, and oesophageal squamous cell carcinoma started to decline, marking the end of the first period and start of the second period.

Frequencies and percentages were calculated for all primary and secondary outcomes. Patient- and tumor characteristics and all primary and secondary outcomes were compared using statistical tests: Chi-square test for categorical variables and Kruskal-Wallis test for continuous variables. A two-sided *p*-value < 0.05 was considered statistically significant.

## Results

A total of 2036 patients undergoing oesophagectomy for oesophageal cancer in Finland during the years 1987–2016 were identified in national registries for inclusion in FINEGO. Patients were excluded due to no cancer or no surgery (19 patients), missing patient records (450 patients), gastric cancer (67 patients), treatment with total gastrectomy (7 patients), and a total of 1493 patients were included in the analyses for the present study. Most of the patients were men (*n* = 1045, 70%) with no comorbidity (*n* = 952, 64%), undergoing open (*n* = 1205, 81%) transthoracic Ivor-Lewis oesophagectomy (*n* = 750, 50%) for pathological stage III (*n* = 556, 38%) oesophageal adenocarcinoma (*n* = 881, 59%). (Table 1)

**Table 1:** Patient- and tumor characteristics of patients undergoing oesophagectomy for oesophageal cancer in Finland in 1987–2016

	1987–1996	1997–2006	2007–2016	Total	
	*n* (%)	*n* (%)	*n* (%)	*n* (%)	*p*-value
**Total**	329 (100.0)	461 (100.0)	703 (100.0)	1493 (100.0)	
**Sex**					**< 0.001**
Male	189 (57.4)	316 (68.5)	540 (76.8)	1045 (70.0)	
Female	140 (42.6)	145 (31.5)	163 (23.2)	448 (30.0)	
**Age**					0.253
Median (IQR)	66 (57.5–72)	64 (56–71)	64 (58–71)	65 (58–71)	
**Tumor stage**					**0.001**
0	3 (1.0)	14 (3.2)	43 (6.2)	60 (4.1)	
I	57 (18.3)	110 (24.8)	185 (26.5)	352 (24.2)	
II	68 (21.9)	70 (15.8)	116 (16.6)	254 (17.5)	
III	131 (42.1)	168 (37.9)	257 (36.8)	556 (38.3)	
IV	38 (12.2)	58 (13.1)	74 (10.6)	170 (11.7)	
Not applicable	14 (4.5)	23 (5.2)	24 (3.4)	61 (4.2)	
**CCI**					**< 0.001**
0	278 (84.5)	303 (65.7)	371 (52.8)	952 (63.8)	
1	40 (12.2)	123 (26.7)	208 (29.6)	371 (24.8)	
2	7 (2.1)	24 (5.2)	82 (11.7)	113 (7.6)	
≥ 3	4 (1.2)	11 (2.4)	42 (6.0)	57 (3.8)	
**Histology**					**< 0.001**
Adenocarcinoma	119 (36.2)	249 (54.0)	513 (73.0)	881 (59.0)	
Squamous cell carcinoma	196 (59.6)	189 (41.0)	166 (23.6)	551 (36.9)	
Other	14 (4.3)	23 (5.0)	24 (3.4)	61 (4.1)	
**Tumor location**					**< 0.001**
Upper	22 (6.7)	12 (2.6)	15 (2.1)	49 (3.3)	
Middle	102 (31.0)	96 (20.8)	99 (14.1)	297 (19.9)	
Lower	142 (43.2)	230 (49.9)	356 (50.6)	728 (48.8)	
GO-junction	63 (19.1)	123 (26.7)	233 (33.1)	419 (28.1)	
**Mode of surgery**					**< 0.001**
Open	329 (100.0)	457 (99.1)	419 (59.6)	1205 (80.7)	
Hybrid	0 (0.0)	2 (0.4)	57 (8.1)	59 (4.0)	
tMIO	0 (0.0)	2 (0.4)	227 (32.3)	229 (15.3)	
**Type of resection**					**< 0.001**
TT Ivor-Lewis	78 (23.7)	190 (41.2)	482 (68.6)	750 (50.2)	
TT McKeown	59 (17.9)	143 (31.0)	118 (16.8)	320 (21.4)	
Transhiatal	159 (48.3)	105 (22.8)	86 (12.2)	350 (23.4)	
Left thoracoabdominal	13 (4.0)	2 (0.4)	1 (0.1)	16 (1.1)	
Proximal gastrectomy	2 (0.6)	1 (0.2)	2 (0.3)	5 (0.3)	
Combined gastro-oesophagectomy	18 (5.5)	20 (4.3)	14 (2.0)	52 (3.5)	
**Conduit material**					**< 0.001**
Stomach	265 (80.5)	404 (87.6)	674 (95.9)	1343 (90.0)	
Small intestine (incl. Roux-en-Y)	28 (8.5)	18 (3.9)	10 (1.4)	56 (3.8)	
Colon	32 (9.7)	36 (7.8)	14 (2.0)	82 (5.5)	
Unclear or other	4 (1.2)	3 (0.7)	5 (0.7)	12 (0.8)	
**Location of anastomosis**					**< 0.001**
Neck	222 (67.5)	247 (53.6)	194 (27.6)	663 (44.4)	
Thorax	105 (31.9)	211 (45.8)	507 (72.1)	823 (55.1)	
Unclear or no anastomosis	2 (0.6)	3 (0.7)	2 (0.3)	7 (0.5)	
**Resection radicality**					**< 0.001**
R0	232 (72.7)	391 (85.9)	634 (90.6)	1257 (85.3)	
R1	52 (16.3)	39 (8.6)	46 (6.6)	137 (9.3)	
R2	35 (11.0)	25 (5.5)	20 (2.9)	80 (5.4)	
**Neoadjuvant treatment**					**< 0.001**
Yes	9 (2.8)	93 (20.4)	400 (57.2)	502 (34.0)	
No	315 (97.2)	362 (79.6)	299 (42.8)	976 (66.0)	

IQR, interquartile range; CCI, Charlson comorbidity index; tMIO, totally minimally invasive oesophagectomy; GO, gastro-oesophageal; TT, transthoracic

Statistical significance is indicated by **bold font**

### Trends in patient- and tumor characteristics

From the years 1987–1996 to 2007–2016, the proportion of males undergoing oesophagectomy increased from 57 to 77% (*p* < 0.001). The average patient in the latest period compared to the earliest period had less advanced disease (37% vs. 42%, stage III, *p* = 0.001), more comorbidities (47% vs. 16%, CCI 1 or more, *p* < 0.001), more adenocarcinoma (73% vs. 36%, *p* < 0.001) and a more distal tumor location (51% vs. 43% lower oesophagus, 33% vs. 19% GO-junction, *p* < 0.001). The operative approach shifted from 100% open surgery in 1987–1996 to 60% open surgery in 2007–2016. The preferred type of resection changed from transhiatal oesophagectomy (48%) to transthoracic Ivor-Lewis oesophagectomy (69%). The use of neoadjuvant treatment increased from 3 to 57%, and the radicality of resection (R0 rate) improved from 73 to 91%. (Table 1)

### Operative outcomes

**Fig. 1:**
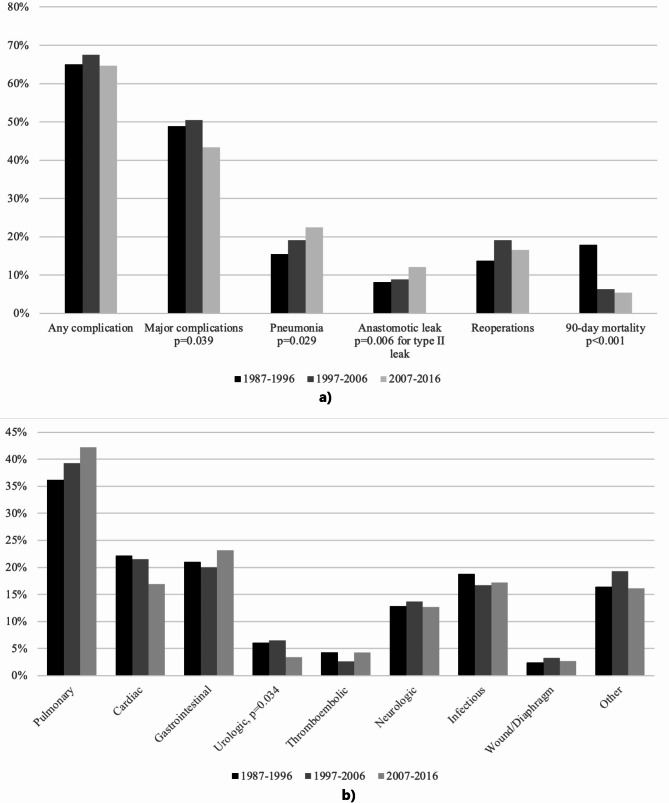
Outcomes of patients undergoing oesophagectomy, stratified by time period **a**. Complication categories defined by ECCG, stratified by time period **b**. Statistically significant *p*-values are shown

Patients undergoing oesophagectomy in 1987–1996, 1997–2006 and 2007–2016 experienced complication rates of 65%, 67.5% and 64.7%, respectively. The most common complications were pneumonia (15.5%, 19.1% and 22.5%, *p* = 0.029) and pleural effusion requiring additional drainage (16.4%, 20.6% and 22.5%, *p* = 0.080), followed by atrial dysrhythmia (15.8%, 17.8% and 15.1%, *p* = 0.464). Anastomotic leakage affected 8.2%, 8.9% and 12.1% of patients, and there was a statistically significant difference in type II leakage across time periods (*p* = 0.006). Conduit necrosis occurred in 2.4%, 2.2% and 3.6% of patients during the three time periods. The most common complication categories defined by ECCG were pulmonary (36.2%, 39.3% and 42.2%, *p* = 0.151), gastrointestinal (21.0%, 20.0% and 23.2%, *p* = 0.398), infectious (18.8%, 16.7% and 17.2%, *p* = 0.722) and cardiac (22.2%, 21.5% and 16.9%, *p* = 0.060) complications. (Table 2; Fig. 1)

**Table 2:** 90-day outcomes of patients undergoing oesophagectomy for oesophageal cancer in Finland in 1987–2016

	1987–1996	1997–2006	2007–2016	
	*n* (%)	*n* (%)	*n* (%)	*p*-value
**Patients**	329 (100.0)	461 (100.0)	703 (100.0)	
**Complications defined by ECCG**				
*Pulmonary*	119 (36.2)	181 (39.3)	298 (42.2)	0.151
Pneumonia	51 (15.5)	88 (19.1)	158 (22.5)	**0.029**
Pleural effusion requiring additional drainage procedure	54 (16.4)	95 (20.6)	158 (22.5)	0.080
Pneumothorax requiring treatment	5 (1.5)	12 (2.6)	23 (3.3)	0.265
Atelectasis mucous plugging requiring bronchoscopy	26 (7.9)	44 (9.5)	51 (7.3)	0.371
Respiratory failure requiring reintubation	48 (14.6)	53 (11.5)	55 (7.8)	**0.003**
Acute aspiration	14 (4.3)	13 (2.8)	25 (3.6)	0.549
Acute respiratory distress syndrome	8 (2.4)	15 (3.3)	20 (2.8)	0.791
Tracheobronchial injury	1 (0.3)	6 (1.3)	2 (0.3)	0.066
Chest tube maintenance for air leak for > 10 d postoperatively	0 (0.0)	1 (0.2)	6 (0.9)	0.110
*Cardiac*	73 (22.2)	99 (21.5)	119 (16.9)	0.060
Cardiac arrest requiring CPR	14 (4.3)	4 (0.9)	8 (1.1)	**< 0.001**
Myocardial infarction	6 (1.8)	11 (2.4)	2 (0.3)	**0.004**
Dysrhythmia atrial requiring treatment	52 (15.8)	82 (17.8)	106 (15.1)	0.464
Dysrhythmia ventricular requiring treatment	2 (0.6)	9 (2.0)	3 (0.4)	**0.024**
Congestive heart failure requiring treatment	12 (3.6)	11 (2.4)	12 (1.7)	0.158
Pericarditis requiring treatment	0 (0.0)	0 (0.0)	0 (0.0)	-
*Gastrointestinal*	69 (21.0)	92 (20.0)	163 (23.2)	0.398
Anastomotic leak	27 (8.2)	41 (8.9)	85 (12.1)	
Type 1	7 (2.1)	12 (2.6)	12 (1.7)	0.576
Type 2	7 (2.1)	11 (2.4)	38 (5.4)	**0.006**
Type 3	13 (4.0)	18 (3.9)	35 (5.0)	0.613
Conduit necrosis	8 (2.4)	10 (2.2)	25 (3.6)	
Type 1	3 (0.9)	2 (0.4)	8 (1.1)	0.448
Type 2	2 (0.6)	3 (0.7)	1 (0.1)	0.325
Type 3	3 (0.9)	5 (1.1)	16 (2.3)	0.150
Ileus defined as small bowel dysfunction preventing or delaying enteral feeding	2 (0.6)	4 (0.9)	11 (1.6)	0.323
Small bowel obstruction	3 (0.9)	2 (0.4)	1 (0.1)	0.189
Feeding J-tube complication	0 (0.0)	9 (2.0)	18 (2.6)	**0.015**
Pyloromyotomy/pyloroplasty complication	2 (0.6)	3 (0.7)	8 (1.1)	0.576
*Clostridium difficile* infection	3 (0.9)	1 (0.2)	8 (1.1)	0.220
Gastrointestinal bleeding requiring intervention or transfusion	7 (2.1)	5 (1.1)	11 (1.6)	0.501
Delayed conduit emptying requiring intervention or delaying discharge or requiring maintenance of NG drainage > 7d postoperatively	6 (1.8)	5 (1.1)	12 (1.7)	0.627
Pancreatitis	2 (0.6)	3 (0.7)	1 (0.1)	0.325
Pancreatic fistula	1 (0.3)	0 (0.0)	2 (0.3)	0.510
Liver dysfunction	1 (0.3)	2 (0.4)	1 (0.1)	0.636
Biliary leakage	0 (0.0)	0 (0.0)	2 (0.3)	0.325
*Urologic*	20 (6.1)	30 (6.5)	24 (3.4)	**0.034**
Acute renal insufficiency (defined as doubling of baseline creatinine)	10 (3.0)	18 (3.9)	16 (2.3)	0.273
Acute renal failure requiring dialysis	0 (0.0)	2 (0.4)	3 (0.4)	0.492
Urinary tract infection	10 (3.0)	11 (2.4)	5 (0.7)	**0.013**
Urinary retention requiring reinsertion of urinary catheter, delaying discharge, or discharge with urinary catheter	2 (0.6)	4 (0.9)	3 (0.4)	0.636
*Thromboembolic*	14 (4.3)	12 (2.6)	30 (4.3)	0.296
Deep venous thrombosis	5 (1.5)	4 (0.9)	3 (0.4)	0.183
Pulmonary embolus	6 (1.8)	8 (1.7)	26 (3.7)	0.071
Stroke (CVA)	3 (0.9)	2 (0.4)	3 (0.4)	0.571
Peripheral thrombophlebitis	0 (0.0)	0 (0.0)	1 (0.1)	0.570
*Neurologic*	42 (12.8)	63 (13.7)	89 (12.7)	0.874
Recurrent nerve injury	19 (5.8)	33 (7.2)	46 (6.5)	
Type 1	18 (5.5)	33 (7.2)	44 (6.3)	0.625
Type 2	0 (0.0)	0 (0.0)	1 (0.1)	0.570
Type 3	1 (0.3)	0 (0.0)	1 (0.1)	0.514
Other neurologic injury	4 (1.2)	5 (1.1)	13 (1.8)	0.518
Acute delirium	21 (6.4)	30 (6.5)	32 (4.6)	0.276
Delirium due to alcohol withdrawal	1 (0.3)	2 (0.4)	1 (0.1)	0.636
*Infectious*	62 (18.8)	77 (16.7)	121 (17.2)	0.722
Wound infection requiring opening wound or antibiotics	16 (4.9)	31 (6.7)	25 (3.6)	**0.048**
Central IV-line infection requiring removal or antibiotics	4 (1.2)	13 (2.8)	8 (1.1)	0.070
Intra-abdominal abscess	9 (2.7)	5 (1.1)	14 (2.0)	0.230
Intrathoracic abscess	20 (6.1)	19 (4.1)	44 (6.3)	0.267
Sepsis defined as positive blood culture	20 (6.1)	20 (4.3)	29 (4.1)	0.357
Other infections requiring antibiotics	9 (2.7)	12 (2.6)	36 (5.1)	**0.046**
*Wound/Diaphragm*	8 (2.4)	15 (3.3)	19 (2.7)	0.766
Thoracic wound dehiscence	0 (0.0)	3 (0.7)	6 (0.9)	0.253
Abdominal wall wound dehiscence	7 (2.1)	9 (2.0)	13 (1.8)	0.955
Acute abdominal wall hernia	1 (0.3)	2 (0.4)	1 (0.1)	0.636
Acute diaphragmatic hernia	1 (0.3)	2 (0.4)	0 (0.0)	0.242
*Other*	54 (16.4)	89 (19.3)	113 (16.1)	0.332
Chyle leak	1 (0.3)	22 (4.8)	38 (5.4)	
Type 1	0 (0.0)	6 (1.3)	5 (0.7)	0.108
Type 2	1 (0.3)	5 (1.1)	19 (2.7)	**0.010**
Type 3	0 (0.0)	11 (2.4)	14 (2.0)	**0.024**
Reoperation for other reasons than bleeding, anastomotic leak or conduit necrosis	27 (8.2)	62 (13.4)	70 (10.0)	0.269
Multiple organ dysfunction syndrome	16 (4.9)	13 (2.8)	7 (1.0)	**0.001**
**Any complication**	214 (65.0)	311 (67.5)	455 (64.7)	0.609
**Major complications**	161 (48.9)	233 (50.5)	305 (43.4)	**0.039**
**Reoperations**	45 (13.7)	88 (19.1)	117 (16.6)	0.113
**ICU stay**,** median (IQR)**	3 (1–6)	2.5 (1–6)	2 (1–4)	**< 0.001**
**Hospital stay**,** median (IQR)**	19 (15–24)	16 (14–22)	14 (11–21)	**< 0.001**
**90-day mortality**	59 (17.9)	29 (6.3)	38 (5.4)	**< 0.001**
**Complication severity**				**< 0.001**
Grade I or no complications	115 (35.0)	150 (32.5)	248 (35.3)	
Grade II	53 (16.1)	78 (16.9)	150 (21.3)	
Grade III	61 (18.5)	129 (28.0)	168 (23.9)	
Grade IV	52 (15.8)	83 (18.0)	114 (16.2)	
Grade V	48 (14.6)	21 (4.6)	23 (3.3)	

ECCG, Esophagectomy Complications Consensus Group; CPR, cardiopulmonary resuscitation; NG, nasogastric; CVA, cerebrovascular accident; IV; intravenous

Statistical significance is indicated by **bold font**

Improvements were seen in several outcomes during the study period. 90-day mortality decreased significantly (17.9%, 6.3% and 5.4%, *p* < 0.001). Major complications decreased, occurring in 48.9%, 50.5% and 43.4% of patients (*p* = 0.039). The median length of ICU-stay decreased from 3 (1–6 interquartile range; IQR), to 2.5 (1–6, IQR), to 2 days (1–4, IQR), *p* < 0.001, and the median hospital stay from 19 (15–24, IQR), to 16 (14–22, IQR), to 14 days (11–21, IQR), *p* < 0.001. Respiratory failure requiring reintubation (*p* = 0.003), cardiac arrest requiring CPR (*p* < 0.001), and multiple organ dysfunction syndrome (*p* = 0.001) were decreased. In complication categories, urologic (6.1%, 6.5% and 3.4%, *p* = 0.034) complications decreased over time. (Table 2; Fig. 1)

Some adverse outcomes also increased over time. Pneumonia and anastomotic leakage were increased. The incidence of reoperations was higher in the latter periods with 13.7%, 19.1% and 16.6% of patients undergoing a reoperation, although this increase was not statistically significant. (Table 2; Fig. 1)

## Discussion

The present study reports the national incidence of 90-day surgical complications during three ten-year periods in Finland. To date, no other studies using standardized definitions have reported time trends in complications after oesophagectomy. Overall, 66% of patients experienced complications, the most common of which were pneumonia, pleural effusion requiring drainage, atrial dysrhythmia and anastomotic leakage.

The main strength of this study was its population-based design, reducing selection bias. The FINEGO-cohort is highly complete in recognition of patients, and the assessment of all clinical variables by expert surgeons allowed accurate data for the analyses. The main weakness was the retrospective design, susceptible to missing data in the earliest years of the study period. Difficulties were introduced by the long study period extending to the 1980s, with some patients having missing records due to them being discarded before the start of data accrual. Finnish legislation allows for discarding of patient records 10 years after the patient’s death. Other weaknesses were the lack of information on clinical staging methods and neoadjuvant treatment patterns, and the lack of data on patients treated after 2016.

The hypothesis of this study was that the introduction of minimally invasive oesophagectomy (MIO) and improvements in staging, patient selection and perioperative care as well as centralization efforts in Finland during the years would have resulted in lower morbidity in the latest decade of the study period. Improvements were seen in many key outcomes, such as a significant improvement in mortality, decreased major complications and shorter ICU- and hospital stays. However, overall complications remained constant at approximately 65%, and increases were seen in pneumonia and anastomotic leakage over time. Reasons behind this can only be speculated on. It seems that those patients who would have died in the earlier periods have been rescued during the latest period but may then have had major and minor complications, resulting in an increase in these outcomes. In addition, a significantly higher proportion of patients had comorbidities in 2007–2016 compared to 1987–1996, associated with a higher risk of surgical complications and mortality [15]. The increased popularity of neoadjuvant treatment in 2007–2016 could have affected the increased occurrence of pneumonia. It has been hypothesized that neoadjuvant chemoradiotherapy might increase pulmonary complications after surgery by affecting the lung parenchyma, however several reports suggest no effect on surgical complications [16–19]. In turn, factors such as the introduction of MIO in 2007 should have decreased the risk of pneumonia and pulmonary complications, which is not demonstrated in these results [20]. Per meta-analyses by Ryan et al. [21] and Hulscher et al. [22], and a more recent RCT on minimally invasive procedures [23], the transhiatal technique and neck anastomoses are shown to increase the risk of anastomotic leakage and recurrent nerve injury. Despite the significantly reduced use of the transhiatal technique and neck anastomoses over the years, both outcomes were increased instead. A contributor to this result might be the coinciding learning curve of MIO, where performing intrathoracic anastomoses is highly demanding in terms of technique. Further, patients operated in 2007–2016 had less advanced tumors compared to patients in 1987–1996.

Despite the most common complications such as pneumonia and anastomotic leakage increasing, instantly life-threatening outcomes such as respiratory failure requiring reintubation, MODS, cardiac arrest requiring CPR and MI were decreased. This, together with the known advances in care, suggest a bias in reporting and documentation of the less dangerous outcomes in the earliest years of the study period. This was also noticed during the data accrual. Decreased major complications, significantly decreased mortality, and shorter ICU- and hospital stays all suggest improved recovery from surgery and better postoperative health, rather than increased morbidity. These outcomes, however, could also have been improved due to better perioperative care and management of complications: the introduction of measures such as nasogastric tube decompression, early enteral feeding, optimized fluid therapy, conservative chest drain usage, optimized pain management, early mobilization and standardized multidisciplinary care described in modern ERAS protocols might have positively influenced outcomes [24]. 

The landmark study introducing the results of 24 high-volume hospitals participating in the creation of the ECCG framework reported rates of major complications at 31.1%, reoperations at 15.7% and 90-day mortality at 4.5% (2-year study from 2015 to 2016) [3]. The first national results following the ECCG framework were reported from the Dutch Upper gastrointestinal Cancer Audit (DUCA). They reported a 28.9% major complication rate, a 12.9% reoperation rate and a 30-day/in-hospital mortality rate of 2.4%, not reporting 90-day mortality (2-year study from 2016 to 2017) [4]. In the International Oesophago-Gastric Anastomosis Audit (OGAA), an international cohort from 137 centers, the rate of major complications, reoperations and 90-day mortality were 25.5%, 12.0% and 4.5%, respectively (9-month study in 2018) [6]. In the latest 10-year period in FINEGO, the rates of major complications, reoperations and 90-day mortality were 43.4%, 16.6% and 5.4%, respectively.

In closer comparison of the national cohorts, the incidence of complications was nearly identical (65% DUCA vs. 64.7% FINEGO). The largest difference was the frequency of major complications. Most of the difference can likely be explained by difference in practice. In FINEGO, pleural effusion requiring additional drainage occurred in 22.5% of patients, compared to 8% in DUCA. As a surgical or radiological intervention, additional drainage is classified as CD grade IIIa, and therefore a major complication. In FINEGO, many patients had an additional drainage procedure as their only complication without other morbidity postoperatively. This describes the practice in Finland to have been more upfront with pleural drainage in comparison, rather than treatment resulting in actual major morbidity more often. This would also explain some of the difference in the pulmonary complication category (33% DUCA vs. 42% FINEGO), as rates of pneumonia (21% DUCA vs. 23% FINEGO) and other pulmonary complications were similar.

Another difference in outcomes was the higher reoperation rate in FINEGO. Reoperations for other reasons than bleeding, anastomotic leak or conduit necrosis were significantly higher in FINEGO at 10% compared to DUCA at 1%, while rates of type III anastomotic leakage (5.1% DUCA vs. 5.0% FINEGO) and type II-III conduit necrosis (0.8% DUCA vs. 2.4% FINEGO) that require surgical interventions were relatively similar. The other reoperations in FINEGO were mainly sequelae of anastomotic leak, such as evacuations of intrathoracic abscesses and tracheostomies, often occurring after 30 days of surgery. Due to complications being assessed for 90 days in FINEGO (compared to 30 days in DUCA), these reoperations were collected, and in turn might have been missed in DUCA, explaining some of the difference between the cohorts. Further, DUCA had a higher incidence of combined leak and necrosis (19%) than FINEGO (15.7%). Type III leak rates were similar between cohorts, and therefore type I-II leak rates were higher in DUCA. This might reflect the longer study period of 10 years in the present study and explain some of the difference in reoperations, as leakage management shifted towards more conservative strategies than surgery at the time.

A significant difference in the characteristics of DUCA and FINEGO was the timeframe of recording of complications: DUCA reported 30-day outcomes, compared to the 90-day outcomes in FINEGO, resulting in higher frequencies of complications. There are also some notable differences in patient- and tumor characteristics as well as treatment approach. Patients in FINEGO had more pathological stage IV tumors (1.3% DUCA vs. 12% FINEGO). Regarding the operative approach, MIO was used in 86% of cases in DUCA, compared to 40% in FINEGO in 2007–2016. Our recent study investigating MIO compared to open oesophagectomy in national practice in Finland found MIO associated with significantly reduced postoperative morbidity, namely major complications, reoperations and pneumonia [25]. These results were in line with randomized evidence to date [20, 26, 27]. The lesser use of MIO in FINEGO compared to DUCA might have affected some of these outcomes in the favor of DUCA. In addition, MIO was implemented in Finland during the time period in question, whereas surgeons performing MIO in the DUCA cohort might have already been beyond their learning curves, as discussed by van der Werf et al. [4] Another factor potentially affecting outcomes is center volume. In the DUCA study, 9 hospitals out of 22 performed 40 or more oesophagectomies, and 5 hospitals 20 or fewer. In FINEGO, there were 20 hospitals performing esophagectomies, and only three of them had at least 20 oesophagectomies annually. The association between higher annual center (and surgeon) volume and better short-term outcomes in complex elective surgery such as oesophagectomy has been well demonstrated [28–30]. 

In turn, the transhiatal approach and neck anastomoses were more frequent in DUCA than FINEGO, associated with higher risk of leakage and recurrent nerve injury [21–23]. Patients in DUCA had also more comorbidities (47% DUCA vs. 53% FINEGO, CCI 0) and a higher frequency of neoadjuvant treatment (93% DUCA vs. 57% FINEGO), which might have affected outcomes.

To conclude, it seems that the developments in oesophageal cancer care during the study period have resulted in less postoperative deaths, which in turn might have increased the relative number of certain complications. Overall, the complication rates and complication profile are comparable to those of DUCA, adding confirmation to existing knowledge on oesophagectomy as an intervention of high postoperative morbidity in national practice. Future nationwide studies reporting outcomes adhering to the ECCG framework are needed to monitor the quality of care provided to patients, as well as to broaden international comparison, better determine realistic baselines for outcomes in national practice and to detect significant differences in outcomes requiring quality improvement measures.

## Data Availability

Research data can be shared for research purposes upon request by contacting the Chief Investigator, Professor Joonas Kauppila, but may be restricted by and require additional permissions from the ethical committee and relevant original data holders.
